# Interactions of the Mechanosensitive Channels with Extracellular Matrix, Integrins, and Cytoskeletal Network in Osmosensation

**DOI:** 10.3389/fnmol.2017.00096

**Published:** 2017-04-05

**Authors:** Runsheng Jiao, Dan Cui, Stephani C. Wang, Dongyang Li, Yu-Feng Wang

**Affiliations:** ^1^Department of Physiology, School of Basic Medical Sciences, Harbin Medical UniversityHarbin, China; ^2^Department of Internal Medicine, Albany Medical CollegeAlbany, NY, USA

**Keywords:** cytoskeleton, extracellular matrix, integrin, transient receptor potential canonical channel, transient receptor potential vallinoid channel, vasopressin

## Abstract

Life is maintained in a sea water-like internal environment. The homeostasis of this environment is dependent on osmosensory system translation of hydromineral information into osmotic regulatory machinery at system, tissue and cell levels. In the osmosensation, hydromineral information can be converted into cellular reactions through osmoreceptors, which changes thirst and drinking, secretion of antidiuretic vasopressin (VP), reabsorption of water and salt in the kidneys at systemic level as well as cellular metabolic activity and survival status at tissue level. The key feature of osmosensation is the activation of mechanoreceptors or mechanosensors, particularly transient receptor potential vallinoid (TRPV) and canonical (TRPC) family channels, which increases cytosolic Ca^2+^ levels, activates osmosensory cells including VP neurons and triggers a series of secondary reactions. TRPV channels are sensitive to both hyperosmotic and hyposmotic stimuli while TRPC channels are more sensitive to hyposmotic challenge in neurons. The activation of TRP channels relies on changes in cell volume, membrane stretch and cytoskeletal reorganization as well as hydration status of extracellular matrix (ECM) and activity of integrins. Different families of TRP channels could be activated differently in response to hyperosmotic and hyposmotic stimuli in different spatiotemporal orders, leading to differential reactions of osmosensory cells. Together, they constitute the osmosensory machinery. The activation of this osmoreceptor complex is also associated with the activity of other osmolarity-regulating organelles, such as water channel protein aquaporins, Na-K-2Cl cotransporters, volume-sensitive anion channels, sodium pump and purinergic receptors in addition to intercellular interactions, typically astrocytic neuronal interactions. In this article, we review our current understandings of the composition of osmoreceptors and the processes of osmosensation.

Since the concept of homeostasis of internal environment was introduced by Claude Bernard and Walter B. Cannon about 100 years ago (Modell et al., 2015), the importance of hydromineral balance in life processes has been extensively explored. However, the identity of osmoreceptors that can detect changes in hydromineral balance and initiate osmotic regulation remains elusive. In this article, we review our current understandings of this osmosensory machinery.

## Osmosensation

Osmosensation requires functioning of osmoreceptors that detect hydromineral disturbance and initiate osmoregulation (Knepper et al., 2015). In the CNS, hypothalamic magnocellular vasopressin (VP) neurons in the supraoptic (SON), paraventricular (PVN), and their accessory magnocellular nuclei (Rhodes et al., 1981) and neurons in the circumventricular organs are considered as the main components of central osmosensory system (McKinley et al., 2004). The central osmosensation involves local neuronal activity, astrocytic plasticity, blood-borne factors, direct osmotic stimuli and autoregulation (Scott and Brown, 2010; Wang et al., 2011; Pedrino et al., 2014); however, the essential requirements for osmosensation are still the ability of osmosensory cells to sense hydromineral changes.

Noteworthy is that other neurons outside of this central osmosensory system can also sense changes in osmotic pressure, such as oxytocin neurons in the SON and PVN (Kortus et al., 2016) and hippocampal neurons (Arranz et al., 2014). In addition, cognitive activity of the cerebrum can exert anticipatory regulation of VP neuronal activity during drinking (Mandelblat-Cerf et al., 2017). In peripheral sites, many types of tissues and cells have the capacity of osmosensation (Pedrino et al., 2014), typically seen in the digestive tract (Zhu et al., 2001) that could change VP neuronal activity through medulla-mediated viscerosensory inputs (Rinaman, 2007). Thus, osmosensation is likely a universal feature among different tissues/cells.

The activation of osmosensory system can change thirst and drinking, secretion of antidiuretic VP, and reabsorption of water and salt in the kidneys (Wang et al., 2011; Danziger and Zeidel, 2015) as well as VP gene transcription following increase in cAMP (Arima et al., 2001), cellular metabolic activity and survival status (Moeckel et al., 2006; Hollborn et al., 2015), thereby helping the body and its parts to restore hydromineral balance.

## Major Cellular Events Evoked by Osmotic Stress

Osmosensation is closely associated with the following cellular events.

### Electrochemical Events

Early studies showed that hyperosmotic stress activates stretch-inactivated cation channels (SICs) and increases the excitability of VP neurons (Voisin and Bourque, 2002). In contrast, short hyposmotic stimulation inactivates the SICs, hyperpolarizes VP neurons and thus reduces VP secretion (Kusano et al., 1999). Further studies showed that the SICs are associated with a class of transient receptor potential (TRP) vallinoid (TRPV) channels since TRPV1- (Sharif Naeini et al., 2006) and TRPV4-null mice (Liedtke and Friedman, 2003) showed reduced hyperosmotic reactions in the organum vasculosum of lamina terminalis.

Further studies reveal that these SICs could also be stretch-activated cation channels because hyposmotic challenges can increase intracellular Ca^2+^ concentration through activation of TRPV1, TRPV2, TRPV4 in Merkel cells from hamster buccal mucosa (Soya et al., 2014), TRPV4 in acinar cells (Aure et al., 2010) and in nonpigmented epithelial cells (Jo et al., 2016). Consistently, in acute hyponatremic condition, serum VP levels increase significantly following initial inhibition (Yagil and Sladek, 1990), which reflects a reactivation of VP neurons following the initial inhibition (Wang et al., 2013a,b) through the mechanism of “resetting osmosensory threshold at the local neural circuit” (Wang et al., 2011). Clearly, the activation of these TRPV channels could occur under both hyperosmotic and hyposmotic challenges.

In fact, many other ion channels are also involved in osmosensation, such as TMEM63 proteins found in *Arabidopsis* (Zhao et al., 2016), TRP ankyrin-1 and TRP melastatin-8 channels in Merkel cells from hamster buccal mucosa (Soya et al., 2014) and P2X receptors that are membrane ion channels gated by extracellular ATP (Fountain et al., 2007). Among them, TRP canonical (TRPC) 5 channel (Jemal et al., 2014) and TRPC6 (Wilson and Dryer, 2014) were found to sense hyposmotic stretch but not hyperosmotic stimulus. Thus, many TRP channels are involved in and play dual role in osmosensation and thus are not specifically bound to hyperosmotic or hyposmotic stimulus; however, TRPC could be more selective to hyposmotic challenge.

### Plasticity of Cytoskeletal Elements

**Figure 1:**
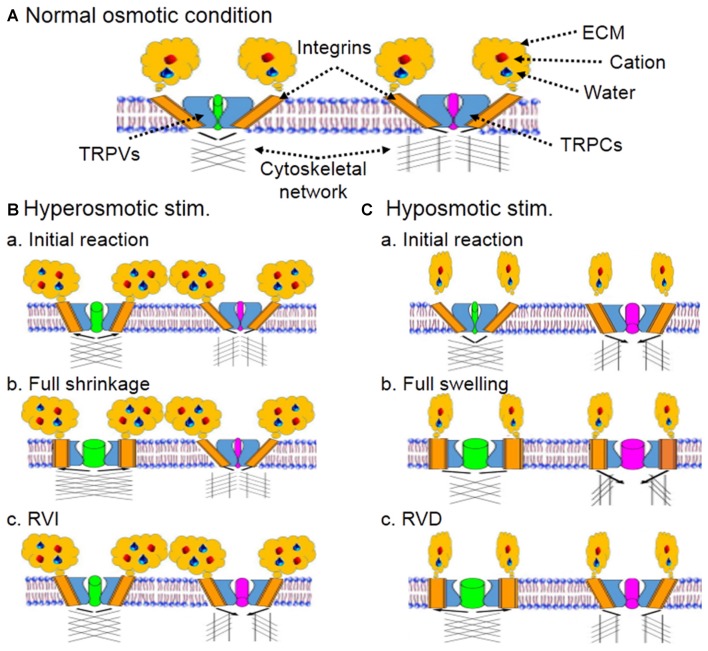
**Schematic diagram of hypothetical osmoreceptors and osmosensation. (A)** Composition of osmoreceptors and osmosensation at resting condition. Cation along with water binds with extracellular matrix (ECM) that interacts with integrins embedded in plasma membrane and spatially-conjugated with transient receptor potential (TRP) vallinoid (TRPV) and canonical (TRPC) family channels. The ECM-integrin-TRP channel complex could bind to microtubule network directly or through actin filaments. The integrins and cytoskeletal networks connected with TRPVs and TRPCs could be different, which would allow hyperosmotic cell shrinkage and hyposmotic swelling to activate the two families in different manners. **(B)** Hyperosmotic stimulus (stim.). **(Ba)** Initial cellular reactions. The ECM binding with cation and water activates TRPV-associated integrins and the ensuing conformational change of integrins leads to partial opening of TRPVs. However, the integrin subunits binding to TRPCs could be different from that to TRPVs and show no activation during cell shrinkage. **(Bb)** Cellular reactions toward full cell shrinkage. Hyperosmotic environment draws water outflow from intracellular compartment, decreases cell volume and increases the pushing force (black arrows) of cytoskeletal network for the full opening of TRPVs. **(Bc)** Regulatory volume increase (RVI) following full cell shrinkage. Activation of TRPVs triggers oscillatory cytosolic Ca^2+^ increase, activates mitogen-activated kinases, and installs more aquaporins on the membrane, thereby leading to a RVI, which could reduce microtubule-associated TRPV opening but cause partial opening of TRPCs through relative volemic increase that yields a pulling force between TRPCs and cytoskeletal network. As a result, hyperosmotic activation of osmosensory neurons occurs. **(C)** Hyposmotic stimulus. **(Ca)** Initial cellular reactions. Hyposmotic environment decreases the activity of integrins by uncoupling ECM with integrins but initiates cell swelling, leading to complete inhibition of TRPVs. The swelling and mild membrane tension causes partial activation of TRPCs, which could result from a pulling force (black arrows) of cytoskeletal network. At this stage, weakly increased cytosolic Ca^2+^ through TRPCs could activate K^+^ current and thus, inhibition of osmosensory neurons occurs. **(Cb)** Cellular reactions toward full cell swelling. As water increasingly gets into the cells and increases intracellular volume, increased interactions between ECM and TRPV-associated integrins cause activation of TRPVs while further activating TRPCs. As a result, reversal of hyposmotic inhibition occurs. **(Cc)** Regulatory volume decrease (RVD) following full cell swelling. As the swelling of cells proceeds, volume-/stretch-sensitive anion channels are also activated, which leads to RVD and volume reduction. Once the RVD occurs, interactions between microtubule networks and TRPVs also increase while opening of TRPCs is partially decreased. As a result, osmosensory neurons could show prolonged excitation or apoptotic alteration.

Cytoskeletal elements including actin filament and microtubule have direct molecular association with the C-terminus of TRPV4 revealed in co-immunoprecipitation (Goswami et al., 2010), and thus could be important regulator of TRP channel activity in osmosensation. Blocking actin polymerization (Prager-Khoutorsky and Bourque, 2010) or disrupting microtubule network (Prager-Khoutorsky and Bourque, 2015) can block hyperosmolarity-evoked activation of osmosensory neurons in rat brain slices. Thus, an increased interactions between microtubule network with TRPV1 during cell shrinkage could account for hyperosmotic activation of osmosensory neurons (Prager-Khoutorsky and Bourque, 2015). However, this hypothesis could not explain hyposmotic intracellular Ca^2+^ increase (Aure et al., 2010; Soya et al., 2014; Jo et al., 2016), the recovery of VP neuronal activity from hyposmotic inhibition (Wang et al., 2013a,b) and the increased VP secretion during volemic increase in chronic osmotic stress (Zhang et al., 2001). Here, referring to the hearing mechanism (Sukharev and Corey, 2004; Martinac, 2014), we propose that if hyperosmotic activation of TRP channels is due to a “push” of microtubule network (Prager-Khoutorsky and Bourque, 2015), the hyposmotic activation of TRP channels should be because of a “pull” of the network in coordination with conformational changes in other cellular components (Figure 1A).

## Cellular Volume

Change in cell volume is a common and remarkable phenomenon in response to hydromineral disturbance. Hyperosmotic stress evokes cell shrinkage which could be followed by a regulatory volume increase (RVI) while hyposmotic challenge causes cell swelling before a regulatory volume decrease (RVD) occurs, which have been shown in cultured neurons (Zhang et al., 2001), astrocytes (Eriksson et al., 1992; Evanko et al., 2004) and hepatocytes (Mundinger et al., 2012). Hence, we further analyze the contribution of these volume changes to osmosensation.

Change in cell volume is an essential driving force of osmosensation. In neurons, progressive decline in cell volume leads to increase in neuronal activity (Figures 1Ba,Bb), and effects of shrinking evoked by mechanical aspiration are quantitatively equivalent to that of hyperosmotic stress in mouse organum vasculosum of lamina terminalis neurons (Ciura et al., 2011). Consistently, cell shrinkage evoked with sucrose or sorbitol can also activate Swiss 3T3 cells (Lunn and Rozengurt, 2004). By contrast, cell swelling or membrane stretch following hyposmotic challenge can also activate TRP channels (Figures 1Ca,Cb), such as TRPV2 in freshly isolated cells from mouse aorta (Muraki et al., 2003), TRPV1, TRPV2 and TRPV4 in mouse odontoblasts (Sato et al., 2013), etc. Consistently, TRPC5 channels of sensory neurons can also be activated by intracellular application of steps of positive pressure through the patch pipette in the whole-cell configuration (Gomis et al., 2008). Noteworthy is that transient hyposmotic Ca^2+^ increase (Sánchez and Wilkins, 2004) can activate Ca^2+^-activated K^+^ channels and that results in inhibition of cellular activity (Ohbuchi et al., 2010) before the occurrence of RVD. Thus, cell volume change could account for osmosensation; however, their excitation or inhibition should be controlled by factors other than volume change itself.

The volume change, ion channel activity and cellular signaling process can form a feedback loop. For example, RVD depends on cation influx following membrane tension during hyposmotic cell swelling as shown in astrocytes and kidney cells (Hua et al., 2010). The influx of Ca^2+^ in turn triggers a series of cellular signaling events, such as activation mitogen-activated protein kinases (MAPKs) including c-Jun NH2-terminal kinase, p38 MAPK and extracellular signal-regulated protein kinase 1/2 (Horiguchi et al., 2011; Shi et al., 2011). The last kinase is known to activate TRP channels (Ebner et al., 2006; Dine et al., 2014).

Osmosensation also involves some osmotic/volemic rebalance machineries, such as aquaporin (AQP)4 (Benfenati et al., 2011; Mola et al., 2016) that mediates water transport and volume alteration, volume-/stretch-sensitive anion channels that release organic osmolytes and Cl^−^ from cytosolic compartment to regain volemic and osmotic balance in swollen cells. Additionally, other membrane transport organelles, such as sodium pumps, Na-K-2Cl cotransporters and sodium-hydrogen exchanges, are also involved in the rebalance of osmolytes across cell membrane (Jia et al., 2016). By changing osmotic gradients, these machineries can sensitize or desensitize the osmoreceptors.

In the CNS, astrocytes are the major target of osmotic disturbance and show remarkable volume changes (Evanko et al., 2004) relative to neurons (Aitken et al., 1998). Importantly, astrocytes can influence neuronal activity through multiple approaches (Wang and Zhu, 2014; Hertz and Chen, 2016; Wang and Parpura, 2016). For instance, β-alanine release from astrocytes (Pasantes-Morales et al., 1994) can inhibit astrocyte GABA transporters and thus inhibits VP secretion through increasing extracellular GABA (Wang et al., 2013a); coordinated D-serine metabolism between astrocytes and magnocellular neurons along with exhaustion of β-alanine and taurine in the SON can increase NMDA receptor activation, and participate in the recovery of VP neurons from hyposmotic inhibition (Wang et al., 2013b). Thus, astrocytes are important osmosensory cells not only by co-expression of TRPV channels and AQP4 (Benfenati et al., 2011; Mola et al., 2016; Iuso and Križaj, 2016) but also by astrocytic plasticity-associated neuronal effects (Hou et al., 2016; Jia et al., 2016).

## Hydration State of Extracellular Matrix (ECM)

Hydrated gel on plasma membrane is the first cellular component to be influenced by hydromineral disturbance. It has been reported that remodeling of glycocalyx can change membrane rafts and the actin cytoskeleton (Zeng and Tarbell, 2014) that in turn modulates TRP channel activity (Prager-Khoutorsky and Bourque, 2015). This hydrated gel has strong binding capacity with cation and water, and thus contributes to osmotic regulation. On cell surface of the SON, there are also abundant polysaccharide-enriched neural cell adhesion molecule and the glycoprotein, tenascin-C (Pierre et al., 2001). These extracellular matrix (ECM) can decrease inter-membrane adhesion mediated by neural cell adhesion molecule (Loers et al., 2014) and thus should share the common effects of ECM on other tissues in osmosensation, such as endothelial cells (Tarbell et al., 2005), cartilage cells (Likhitpanichkul et al., 2005), hepatocytes (Mundinger et al., 2012), kidney cells (Shestopalova et al., 2008) and neurons (Arranz et al., 2014).

We propose that during hyperosmotic stress, ECM binds with excessive cation, buffers osmotic stress, reduces membrane stretch (Figure 1Ba) while accelerating water efflux to reduce intracellular volume (Figure 1Bb), and thus increases the interactions between integrin, actin filament and microtubules (Sims et al., 1992; Martinac, 2014). During hyposmotic challenges, ECM binds with fewer cation and water, which allows excessive water to get into the cell and increases intracellular volume, and thus decreases the pushing/opening force of microtubules on TRPV channels (Prager-Khoutorsky and Bourque, 2015), resulting in stretch-inactivation of TRPVs (Figure 1Ca). These proposals are supported by the finding that in hyaluronan synthase HAS3^−/−^ slices, spontaneous epileptiform activity in the CA1 hippocampus was blocked by hyperosmotic stress (Arranz et al., 2014). This is likely because excessive cation introduced by the hyperosmotic solution, not bound to ECM in HAS3^−/−^ slices, quickly diffuses into intracellular space through the Na-K-2Cl cotransporter 2 (Konopacka et al., 2015) and other osmolyte transport organelles (Jia et al., 2016), and thus increase cellular volume and interrupt the interactions between TRPV channels and cytoskeletal elements. In the same experiment, hyposmotic solution induced spontaneous epileptiform activity in the CA1 hippocampus, which was blocked upon returning to normoosmotic solution (Arranz et al., 2014). The hyposmotic reaction could result from fast water influx and swelling-evoked RVD, while the blocking effect of normoosmotic solution could be due to osmotic restoration of cell volume, similar to the effect of hyperosmotic solution (Arranz et al., 2014).

Considering the involvement of other TRP channels in osmosensation, we further propose that hyposmotic volume increase could increase the pulling force of microtubules on TRPC channels that could increasingly open TRPC channels (Figure 1Cb) following the increases in membrane stretch (Gomis et al., 2008) and compensate for the reduced excitatory effects of closing TRPV channels, resulting in hyposmotic activation of osmosensory cells.

Hydration state of ECM could also function through altering extracellular space (ECS). Without ECM binding in hyaluronan synthase HAS3^−/−^ slices, hyperosmotic stress increased ECS in brain slices by attracting more water efflux in exchange of ion influx, while hyposmotic challenge reduced ECS by promoting more water influx (Arranz et al., 2014). The increased ECS also decreases cellular apposition and reduces inter-neuronal interactions in the CA1 hippocampus, which is a condition known to reduce VP neuronal activity in the SON; the increased cellular apposition due to reduced ECS can increase junctional coupling and mutually excitatory influence between adjacent cells (Tweedle and Hatton, 1977; Tweedle et al., 1993), thereby increasing neuronal activity and their synchronization (Hatton, 1990; Theodosis et al., 2008).

## Integrins

In extracellular to intracellular signal transduction, integrins play a critical role as an ECM receptor in whole body including the hypothalamus (Ablooglu et al., 2007; Gao et al., 2007) and the SON (Seidah et al., 1991). It has been reported that TRPV1 receptor is co-expressed with integrin subunits that bind fibronectin (Jeske et al., 2009); GM1 ganglioside binds to TRPC5 by the mediation of integrin (Wu et al., 2007). Thus, integrins are the mediator of ECM regulation of TRP channel activity in osmosensation.

It is known that active integrin complexes are specifically enriched for proteins associated with microtubule-based functions; active integrins establish an environment to stabilize microtubules at the cell periphery (Byron et al., 2015). Moreover, the activation of membrane integrins elicits actin cytoskeleton reorganization (Jin et al., 2011) via integrin-linked kinase (O’Meara et al., 2013). Thus, osmotic conformational alterations of integrins could change TRP channel activity.

This proposal is supported by the following evidence. Silencing integrin β1 expression reduces RVD of the adherent cells (Sørensen et al., 2015). Interrupting an integrin β3/Src/ClC-3 signaling pathway influences the hyposmotic activation of volume-regulated chloride channels (Zeng et al., 2014). Deletion of the integrin alpha1 subunit inhibited the intracellular Ca^2+^ transients of chondrocytes to hyposmotic stress *ex vivo* and *in vitro* (Jablonski et al., 2014). Thus, integrins are the key components in relaying ECM signals under various osmotic conditions.

Integrin involvement in osmosensation is related to the following approaches (Figure 1A). (1) Contacts between ECM and integrin are a prerequisite of osmotic cellular responses, such as hyperosmotic enhancement of spontaneous quantal release of neurotransmitter (Kashani et al., 2001) and glutamine uptake in response to hyposmotic and hyperosmotic exposure (Low and Taylor, 1998). (2) There are direct interactions between TRPV4, alpha2 integrin and the Src tyrosine kinase Lyn (Alessandri-Haber et al., 2008) and co-expression of the TRPV1 receptor with integrin subunits that bind fibronectin in sensory neurons (Jeske et al., 2009). (3) Activated integrins could stabilize microtubules at the cell periphery (Byron et al., 2015) to form an integrin- TRPV-microtubule complex (Goswami et al., 2010) through eliciting actin cytoskeleton reorganization (Jin et al., 2011).

Under different osmotic conditions, signaling process modulated by integrin-ECM contacts is either activated or inhibited in a time-dependent manner. For example, upon hyposmotic stimulation, more water retains in the interstitial tissues at the initial stage, which gives rise to a disjoining force and places the integrin-ECM bonds under mechanical tension, thus accelerating their dissociation and inactivating integrins (Halperin and Kröger, 2012). Once cell swelling occurs, the increased cell volume pushes membrane out-bound expansion and leads to increases in integrin/ECM contacts, which causes the activation of integrins as shown in the volume regulation of skeletal muscles (Low and Taylor, 1998) and hepatocytes (Mundinger et al., 2012). Resultantly, cells experience an initial inhibition and subsequent activation.

## Concluding Remarks

Osmosensation is a complex cellular process involving coordinated interactions between extracellular and intracellular processes, particularly involving ECM, integrins, actin filament, microtubule and TRP channels. (1) Increased ECM-integrin interactions during hyperosmotic stress could directly activate TRPV channels by conformational change-associated “gate” opening through actin reorganization (Jin et al., 2011). This external signal could work coordinately with increased interactions between microtubule network and TRP channels during hyperosmotic shrinkage (Prager-Khoutorsky and Bourque, 2015) to push TRP channel opening (Figure 1B). (2) Prolonged hyperosmotic stress promotes VP gene transcription and translation and RVI (Figure 1Bc) while maintaining ECM-integrin-associated gate opening. (3) Early hyposmotic tension causes mild conformational change in integrins-TRP complex, inhibition of TRPV channels by their uncoupling with microtubule network and the “leakage” of these channels (Muraki et al., 2003; Sato et al., 2013; Jo et al., 2016) including ATP. ATP activation of purine-associated opening of TRPC and other TRP channels (Goel et al., 2007; Soya et al., 2014) can quickly reverse this initial inhibition and lead to cytosolic Ca^2+^ increase. The opening of TRPC channels is likely achieved by a pulling force exerted by actin-microtubule network (Figure 1C). (4) Further cellular swelling increases ECM-integrin interaction and activation of TRPVs while triggering RVD which further strengthens the activation of TRPVs even activity of TRPCs could be reduced (Figure 1Cc). Certainly, the buffer effects of ion-transporting organelles, AQPs, and volume-/stretch-sensitive anion channels on osmotic gradients can modulate the osmosensation by changing the osmotic gradients across the membrane. Moreover, hyposmotic effects on cell volume (Lohr and Yohe, 2000) and VP secretion (Yagil and Sladek, 1990) are largely rate-dependent, and thus the large buffering capacity of the body can account for the resistance of brain to osmotic maladaptation (Go, 1997; Verbalis, 2010).

Further studies should address the structural and functional relationship between ECM-integrin bonding and cytoskeletal elements as well as the temporal association between TRP channel activation and cell volume change during osmotic stimuli in osmosensory neurons. Moreover, the relationship between instant VP release from pre-existing VP pool and delayed transcription of VP gene (Arima et al., 1999, 2010) should be clarified. Worth noting is also that the osmotic responses of SON cells (Tweedle and Hatton, 1977) are much faster and stronger than PVN cells (Gregory et al., 1980). It is interesting to further explore potential differences in their histology and the functioning of the osmoreceptors. Lastly, interactions between local and systemic osmotic factors should be clarified as well. Answering these challenging questions would shed more light on a variety of medical and biological etiologies that are currently not well understood yet.

## Author Contributions

RJ, DC and SCW wrote different sections of the first draft; DL participated in the revision; Y-FW designed the review and made final revision.

## Conflict of Interest Statement

The authors declare that the research was conducted in the absence of any commercial or financial relationships that could be construed as a potential conflict of interest.

## References

[B1] Ablooglu A. J. Kang J. Handin R. I. Traver D. Shattil S. J. (2007). The zebrafish vitronectin receptor: characterization of integrin alphaV and beta3 expression patterns in early vertebrate development. Dev. Dyn. 236, 2268–2276. 10.1002/dvdy.21229 17626277

[B2] Aitken P. G. Borgdorff A. J. Juta A. J. A. Kiehart D. P. Somjen G. G. Wadman W. J. (1998). Volume changes induced by osmotic stress in freshly isolated rat hippocampal neurons. Pflugers Arch. 436, 991–998. 10.1007/s004240050734 9799418

[B3] Alessandri-Haber N. Dina O. A. Joseph E. K. Reichling D. B. Levine J. D. (2008). Interaction of transient receptor potential vanilloid 4, integrin, and SRC tyrosine kinase in mechanical hyperalgesia. J. Neurosci. 28, 1046–1057. 10.1523/JNEUROSCI.4497-07.2008 18234883 PMC6671413

[B4] Arima H. Baler R. Aguilera G. (2010). Fos proteins are not prerequisite for osmotic induction of vasopressin transcription in supraoptic nucleus of rats. Neurosci. Lett. 486, 5–9. 10.1016/j.neulet.2010.09.030 20850504 PMC3408597

[B5] Arima H. House S. B. Gainer H. Aguilera G. (2001). Direct stimulation of arginine vasopressin gene transcription by cAMP in parvocellular neurons of the paraventricular nucleus in organotypic cultures. Endocrinology 142, 5027–5030. 10.1210/en.142.11.5027 11606471

[B6] Arima H. Kondo K. Kakiya S. Nagasaki H. Yokoi H. Yambe Y.. (1999). Rapid and sensitive vasopressin heteronuclear RNA responses to changes in plasma osmolality. J. Neuroendocrinol. 11, 337–341. 10.1046/j.1365-2826.1999.00308.x 10320560

[B7] Arranz A. M. Perkins K. L. Irie F. Lewis D. P. Hrabe J. Xiao F.. (2014). Hyaluronan deficiency due to Has3 knock-out causes altered neuronal activity and seizures via reduction in brain extracellular space. J. Neurosci. 34, 6164–6176. 10.1523/JNEUROSCI.3458-13.2014 24790187 PMC4004806

[B8] Aure M. H. Røed A. Galtung H. K. (2010). Intracellular Ca^2+^ responses and cell volume regulation upon cholinergic and purinergic stimulation in an immortalized salivary cell line. Eur. J. Oral Sci. 118, 237–244. 10.1111/j.1600-0722.2010.00738.x 20572856

[B9] Benfenati V. Caprini M. Dovizio M. Mylonakou M. N. Ferroni S. Ottersen O. P.. (2011). An aquaporin-4/transient receptor potential vanilloid 4 (AQP4/TRPV4) complex is essential for cell-volume control in astrocytes. Proc. Natl. Acad. Sci. U S A 108, 2563–2568. 10.1073/pnas.1012867108 21262839 PMC3038710

[B10] Byron A. Askari J. A. Humphries J. D. Jacquemet G. Koper E. J. Warwood S.. (2015). A proteomic approach reveals integrin activation state-dependent control of microtubule cortical targeting. Nat. Commun. 6:6135. 10.1038/ncomms7135 25609142 PMC4317495

[B11] Ciura S. Liedtke W. Bourque C. W. (2011). Hypertonicity sensing in organum vasculosum lamina terminalis neurons: a mechanical process involving TRPV1 but not TRPV4. J. Neurosci. 31, 14669–14676. 10.1523/JNEUROSCI.1420-11.2011 21994383 PMC6703397

[B12] Danziger J. Zeidel M. L. (2015). Osmotic homeostasis. Clin. J. Am. Soc. Nephrol. 10, 852–862. 10.2215/CJN.10741013 25078421 PMC4422250

[B13] Dine J. Ducourneau V. R. Fénelon V. S. Fossat P. Amadio A. Eder M.. (2014). Extracellular signal-regulated kinase phosphorylation in forebrain neurones contributes to osmoregulatory mechanisms. J. Physiol. 592, 1637–1654. 10.1113/jphysiol.2013.261008 24492838 PMC3979616

[B14] Ebner H. L. Fiechtner B. Pelster B. Krumschnabel G. (2006). Extracellular signal regulated MAP-kinase signalling in osmotically stressed trout hepatocytes. Biochim. Biophys. Acta 1760, 941–950. 10.1016/j.bbagen.2006.03.017 16650600

[B15] Eriksson P. S. Nilsson M. Wågberg M. Rönnbäck L. Hansson E. (1992). Volume regulation of single astroglial cells in primary culture. Neurosci. Lett. 143, 195–199. 10.1016/0304-3940(92)90264-8 1436666

[B16] Evanko D. S. Zhang Q. Zorec R. Haydon P. G. (2004). Defining pathways of loss and secretion of chemical messengers from astrocytes. Glia 47, 233–240. 10.1002/glia.20050 15252812

[B17] Fountain S. J. Parkinson K. Young M. T. Cao L. Thompson C. R. North R. A. (2007). An intracellular P2X receptor required for osmoregulation in Dictyostelium discoideum. Nature 448, 200–203. 10.1038/nature05926 17625565 PMC3942652

[B18] Gao Y.-Z. Guo S.-Y. Yin Q.-Z. Cui X.-Q. Hisamitsu T. Jiang X.-H. (2007). Possible involvement of integrin signaling pathway in the process of recovery from restraint stress in rats. Neurosci. Bull. 23, 229–235. 10.1007/s12264-007-0034-x 17687398 PMC5550586

[B19] Go K. G. (1997). The normal and pathological physiology of brain water. Adv. Tech. Stand. Neurosurg. 23, 47–142. 10.1007/978-3-7091-6549-2_2 9075471

[B20] Goel M. Sinkins W. G. Zuo C. D. Hopfer U. Schilling W. P. (2007). Vasopressin-induced membrane trafficking of TRPC3 and AQP2 channels in cells of the rat renal collecting duct. Am. J. Physiol. Renal Physiol. 293, F1476–F1488. 10.1152/ajprenal.00186.2007 17699554

[B200] Gomis A. Soriano S. Belmonte C. Viana F. (2008). Hypoosmotic- and pressure-induced membrane stretch activate TRPC5 channels. J. Physiol. 586, 5633–5649. 10.1113/jphysiol.2008.161257 18832422 PMC2655392

[B21] Goswami C. Kuhn J. Heppenstall P. A. Hucho T. (2010). Importance of non-selective cation channel TRPV4 interaction with cytoskeleton and their reciprocal regulations in cultured cells. PLoS One 5:e11654. 10.1371/journal.pone.0011654 20657843 PMC2906515

[B22] Gregory W. A. Tweedle C. D. Hatton G. I. (1980). Ultrastructure of neurons in the paraventricular nucleus of normal, dehydrated and rehydrated rats. Brain Res. Bull. 5, 301–306. 10.1016/0361-9230(80)90173-2 7397574

[B23] Halperin A. Kröger M. (2012). Theoretical considerations on mechanisms of harvesting cells cultured on thermoresponsive polymer brushes. Biomaterials 33, 4975–4987. 10.1016/j.biomaterials.2012.03.060 22502791

[B24] Hatton G. I. (1990). Emerging concepts of structure-function dynamics in adult brain: the hypothalamo-neurohypophysial system. Prog. Neurobiol. 34, 437–504. 10.1016/0301-0082(90)90017-b 2202017

[B25] Hertz L. Chen Y. (2016). Importance of astrocytes for potassium ion (K^+^) homeostasis in brain and glial effects of K^+^ and its transporters on learning. Neurosci. Biobehav. Rev. 71, 484–505. 10.1016/j.neubiorev.2016.09.018 27693230

[B26] Hollborn M. Vogler S. Reichenbach A. Wiedemann P. Bringmann A. Kohen L. (2015). Regulation of the hyperosmotic induction of aquaporin 5 and VEGF in retinal pigment epithelial cells: involvement of NFAT5. Mol. Vis. 21, 360–377. 25878490 PMC4390809

[B27] Horiguchi K. Fujiwara K. Ilmiawati C. Kikuchi M. Tsukada T. Kouki T.. (2011). Caveolin 3-mediated integrin β1 signaling is required for the proliferation of folliculostellate cells in rat anterior pituitary gland under the influence of extracellular matrix. J. Endocrinol. 210, 29–36. 10.1530/JOE-11-0103 21508095

[B28] Hou D. Jin F. Li J. Lian J. Liu M. Liu X. (2016). Model roles of the hypothalamo-neurohypophysial system in neuroscience study. Biochem. Pharmacol. 5:211 10.4172/2167-0501.1000211

[B29] Hua S. Z. Gottlieb P. A. Heo J. Sachs F. (2010). A mechanosensitive ion channel regulating cell volume. Am. J. Physiol. Cell Physiol. 298, C1424–C1430. 10.1152/ajpcell.00503.2009 20457830 PMC2889639

[B30] Iuso A. Križaj D. (2016). TRPV4-AQP4 interactions ‘turbocharge’ astroglial sensitivity to small osmotic gradients. Channels 10, 172–174. 10.1080/19336950.2016.1140956 26760501 PMC4954570

[B31] Jablonski C. L. Ferguson S. Pozzi A. Clark A. L. (2014). Integrin α1β1 participates in chondrocyte transduction of osmotic stress. Biochem. Biophys. Res. Commun. 445, 184–190. 10.1016/j.bbrc.2014.01.157 24495803 PMC4022045

[B32] Jemal I. Soriano S. Conte A. L. Morenilla C. Gomis A. (2014). G protein-coupled receptor signalling potentiates the osmo-mechanical activation of TRPC5 channels. Pflugers Arch. 466, 1635–1646. 10.1007/s00424-013-1392-z 24177920

[B33] Jeske N. A. Patwardhan A. M. Henry M. A. Milam S. B. (2009). Fibronectin stimulates TRPV1 translocation in primary sensory neurons. J. Neurochem. 108, 591–600. 10.1111/j.1471-4159.2008.05779.x 19012739 PMC2678239

[B34] Jia S.-W. Liu X.-Y. Wang S. C. Wang Y.-F. (2016). Vasopressin hypersecretion-associated brain edema formation in ischemic stroke: underlying mechanisms. J. Stroke Cerebrovasc. Dis. 25, 1289–1300. 10.1016/j.jstrokecerebrovasdis.2016.02.002 27068863

[B35] Jin M. Berrout J. O’Neil R. G. (2011). “Regulation of TRP channels by osmomechanical stress,” in TRP Channels, ed. Zhu M. X. (Boca Raton, FL: CRC Press), 353–373. 22593965

[B36] Jo A. O. Lakk M. Frye A. M. Phuong T. T. Redmon S. N. Roberts R.. (2016). Differential volume regulation and calcium signaling in two ciliary body cell types is subserved by TRPV4 channels. Proc. Natl. Acad. Sci. U S A 113, 3885–3890. 10.1073/pnas.1515895113 27006502 PMC4833269

[B37] Kashani A. H. Chen B.-M. Grinnell A. D. (2001). Hypertonic enhancement of transmitter release from frog motor nerve terminals: Ca^2+^ independence and role of integrins. J. Physiol. 530, 243–252. 10.1111/j.1469-7793.2001.0243l.x 11208972 PMC2278411

[B38] Knepper M. A. Kwon T. H. Nielsen S. (2015). Molecular physiology of water balance. N. Engl. J. Med. 373:196. 10.1056/NEJMc1505505 26154805

[B39] Konopacka A. Qiu J. Yao S. T. Greenwood M. P. Greenwood M. Lancaster T.. (2015). Osmoregulation requires brain expression of the renal Na-K-2Cl cotransporter NKCC2. J. Neurosci. 35, 5144–5155. 10.1523/JNEUROSCI.4121-14.2015 25834041 PMC4380993

[B40] Kortus S. Srinivasan C. Forostyak O. Ueta Y. Sykova E. Chvatal A.. (2016). Physiology of spontaneous [Ca^2+^]i oscillations in the isolated vasopressin and oxytocin neurones of the rat supraoptic nucleus. Cell Calcium 59, 280–288. 10.1016/j.ceca.2016.04.001 27072326 PMC4969632

[B41] Kusano K. House S. B. Gainer H. (1999). Effects of osmotic pressure and brain-derived neurotrophic factor on the survival of postnatal hypothalamic oxytocinergic and vasopressinergic neurons in dissociated cell culture. J. Neuroendocrinol. 11, 145–152. 10.1046/j.1365-2826.1999.00296.x 10048470

[B42] Liedtke W. Friedman J. M. (2003). Abnormal osmotic regulation in *trpv4*^−/−^ mice. Proc. Natl. Acad. Sci. U S A 100, 13698–13703. 10.1073/pnas.1735416100 14581612 PMC263876

[B43] Likhitpanichkul M. Guo X. E. Mow V. C. (2005). The effect of matrix tension-compression nonlinearity and fixed negative charges on chondrocyte responses in cartilage. Mol. Cell. Biomech. 2, 191–204. 16705865

[B44] Loers G. Saini V. Mishra B. Papastefanaki F. Lutz D. Chaudhury S.. (2014). Nonyloxytryptamine mimics polysialic acid and modulates neuronal and glial functions in cell culture. J. Neurochem. 128, 88–100. 10.1111/jnc.12408 23957498

[B45] Lohr J. W. Yohe L. (2000). Isovolumetric regulation of rat glial cells during development and correction of hypo-osmolality. Neurosci. Lett. 286, 5–8. 10.1016/s0304-3940(00)01098-3 10822139

[B46] Low S. Y. Taylor P. M. (1998). Integrin and cytoskeletal involvement in signalling cell volume changes to glutamine transport in rat skeletal muscle. J. Physiol. 512, 481–485. 10.1111/j.1469-7793.1998.481be.x 9763637 PMC2231196

[B47] Lunn J. A. Rozengurt E. (2004). Hyperosmotic stress induces rapid focal adhesion kinase phosphorylation at tyrosines 397 and 577. Role of Src family kinases and Rho family GTPases. J. Biol. Chem. 279, 45266–45278. 10.1074/jbc.M314132200 15302877

[B48] Mandelblat-Cerf Y. Kim A. Burgess C. R. Subramanian S. Tannous B. A. Lowell B. B.. (2017). Bidirectional anticipation of future osmotic challenges by vasopressin neurons. Neuron 93, 57–65. 10.1016/j.neuron.2016.11.021 27989461 PMC5215952

[B49] Martinac B. (2014). The ion channels to cytoskeleton connection as potential mechanism of mechanosensitivity. Biochim. Biophys. Acta 1838, 682–691. 10.1016/j.bbamem.2013.07.015 23886913

[B50] McKinley M. J. Mathai M. L. McAllen R. M. McClear R. C. Miselis R. R. Pennington G. L.. (2004). Vasopressin secretion: osmotic and hormonal regulation by the lamina terminalis. J. Neuroendocrinol. 16, 340–347. 10.1111/j.0953-8194.2004.01184.x 15089972

[B51] Modell H. Cliff W. Michael J. McFarland J. Wenderoth M. P. Wright A. (2015). A physiologist’s view of homeostasis. Adv. Physiol. Educ. 39, 259–266. 10.1152/advan.00107.2015 26628646 PMC4669363

[B52] Moeckel G. W. Zhang L. Chen X. Rossini M. Zent R. Pozzi A. (2006). Role of integrin α1β1 in the regulation of renal medullary osmolyte concentration. Am. J. Physiol. Renal Physiol. 290, F223–F231. 10.1152/ajprenal.00371.2004 16106035

[B53] Mola M. G. Sparaneo A. Gargano C. D. Spray D. C. Svelto M. Frigeri A.. (2016). The speed of swelling kinetics modulates cell volume regulation and calcium signaling in astrocytes: a different point of view on the role of aquaporins. Glia 64, 139–154. 10.1002/glia.22921 26413835 PMC4905710

[B54] Mundinger T. A. Sommerfeld A. Reinehr R. Spatz J. P. Häussinger D. Boehm H. (2012). Investigating cell-ECM contact changes in response to hypoosmotic stimulation of hepatocytes *in vivo* with DW-RICM. PLoS One 7:e48100. 10.1371/journal.pone.0048100 23110181 PMC3482193

[B55] Muraki K. Iwata Y. Katanosaka Y. Ito T. Ohya S. Shigekawa M.. (2003). TRPV2 is a component of osmotically sensitive cation channels in murine aortic myocytes. Circ. Res. 93, 829–838. 10.1161/01.RES.0000097263.10220.0C 14512441

[B56] Ohbuchi T. Yokoyama T. Saito T. Suzuki H. Fujihara H. Katoh A.. (2010). Modulators of BK and SK channels alter electrical activity *in vitro* in single vasopressin neurons isolated from the rat supraoptic nucleus. Neurosci. Lett. 484, 26–29. 10.1016/j.neulet.2010.08.010 20708068

[B57] O’Meara R. W. Michalski J.-P. Anderson C. Bhanot K. Rippstein P. Kothary R. (2013). Integrin-linked kinase regulates process extension in oligodendrocytes via control of actin cytoskeletal dynamics. J. Neurosci. 33, 9781–9793. 10.1523/JNEUROSCI.5582-12.2013 23739974 PMC6619710

[B58] Pasantes-Morales H. Murray R. A. Sánchez-Olea R. Morán J. (1994). Regulatory volume decrease in cultured astrocytes. II. Permeability pathway to amino acids and polyols. Am. J. Physiol. 266, C172–C178. 8304414 10.1152/ajpcell.1994.266.1.C172

[B59] Pedrino G. R. Rosa D. A. Lopes O. U. Cravo S. L. (2014). “Catecholaminergic medullary pathways and cardiovascular responses to expanded circulating volume and increased osmolarity,” in Neurobiology of Body Fluid Homeostasis: Transduction and Integration, eds Menani L. A. Jr. De Luca J. V. Johnson A. K. (Boca Raton, FL: CRC Press), 123–140. 24829998

[B60] Pierre K. Bonhomme R. Dupouy B. Poulain D. A. Theodosis D. T. (2001). The polysialylated neural cell adhesion molecule reaches cell surfaces of hypothalamic neurons and astrocytes via the constitutive pathway. Neuroscience 103, 133–142. 10.1016/s0306-4522(00)00536-4 11311794

[B61] Prager-Khoutorsky M. Bourque C. W. (2010). Osmosensation in vasopressin neurons: changing actin density to optimize function. Trends Neurosci. 33, 76–83. 10.1016/j.tins.2009.11.004 19963290

[B62] Prager-Khoutorsky M. Bourque C. W. (2015). Mechanical basis of osmosensory transduction in magnocellular neurosecretory neurones of the rat supraoptic nucleus. J. Neuroendocrinol. 27, 507–515. 10.1111/jne.12270 25712904

[B63] Rhodes C. H. Morrell J. I. Pfaff D. W. (1981). Immunohistochemical analysis of magnocellular elements in rat hypothalamus: distribution and numbers of cells containing neurophysin, oxytocin, and vasopressin. J. Comp. Neurol. 198, 45–64. 10.1002/cne.901980106 7014660

[B64] Rinaman L. (2007). Visceral sensory inputs to the endocrine hypothalamus. Front. Neuroendocrinol. 28, 50–60. 10.1016/j.yfrne.2007.02.002 17391741 PMC1945046

[B65] Sánchez J. C. Wilkins R. J. (2004). Changes in intracellular calcium concentration in response to hypertonicity in bovine articular chondrocytes. Comp. Biochem. Physiol. A Mol. Integr. Physiol. 137, 173–182. 10.1016/j.cbpb.2003.09.025 14720602

[B66] Sato M. Sobhan U. Tsumura M. Kuroda H. Soya M. Masamura A.. (2013). Hypotonic-induced stretching of plasma membrane activates transient receptor potential vanilloid channels and sodium-calcium exchangers in mouse odontoblasts. J. Endod. 39, 779–787. 10.1016/j.joen.2013.01.012 23683279

[B67] Scott V. Brown C. H. (2010). State-dependent plasticity in vasopressin neurones: dehydration-induced changes in activity patterning. J. Neuroendocrinol. 22, 343–354. 10.1111/j.1365-2826.2010.01961.x 20088912

[B68] Seidah N. G. Marcinkiewicz M. Benjannet S. Gaspar L. Beaubien G. Mattei M. G.. (1991). Cloning and primary sequence of a mouse candidate prohormone convertase PC1 homologous to PC2, Furin, and Kex2: distinct chromosomal localization and messenger RNA distribution in brain and pituitary compared to PC2. Mol. Endocrinol. 5, 111–122. 10.1210/mend-5-1-111 2017186

[B69] Sharif Naeini R. Witty M. F. Séguéla P. Bourque C. W. (2006). An N-terminal variant of Trpv1 channel is required for osmosensory transduction. Nat. Neurosci. 9, 93–98. 10.1038/nn1614 16327782

[B70] Shestopalova L. V. Lavrinenko V. A. Shkurupiy V. A. Ivanova L. N. (2008). Involvement of interstitial structures of the kidney into hydrosmotic effect of vasopressin (morphofunctional study). Bull. Exp. Biol. Med. 146, 682–686. 10.1007/s10517-009-0372-y 19513354

[B71] Shi Z. D. Wang H. Tarbell J. M. (2011). Heparan sulfate proteoglycans mediate interstitial flow mechanotransduction regulating MMP-13 expression and cell motility via FAK-ERK in 3D collagen. PLoS One 6:e15956. 10.1371/journal.pone.0015956 21246051 PMC3016412

[B72] Sims J. R. Karp S. Ingber D. E. (1992). Altering the cellular mechanical force balance results in integrated changes in cell, cytoskeletal and nuclear shape. J. Cell Sci. 103, 1215–1222. 1487498 10.1242/jcs.103.4.1215

[B73] Sørensen B. H. Rasmussen L. J. H. Broberg B. S. Klausen T. K. Sauter D. P. R. Lambert I. H.. (2015). Integrin β1, osmosensing, and chemoresistance in mouse ehrlich carcinoma cells. Cell. Physiol. Biochem. 36, 111–132. 10.1159/000374057 25925201

[B74] Soya M. Sato M. Sobhan U. Tsumura M. Ichinohe T. Tazaki M.. (2014). Plasma membrane stretch activates transient receptor potential vanilloid and ankyrin channels in Merkel cells from hamster buccal mucosa. Cell Calcium 55, 208–218. 10.1016/j.ceca.2014.02.015 24642224

[B75] Sukharev S. Corey D. P. (2004). Mechanosensitive channels: multiplicity of families and gating paradigms. Sci. STKE 2004:re4. 10.1126/stke.2192004re4 14872099

[B76] Tarbell J. M. Weinbaum S. Kamm R. D. (2005). Cellular fluid mechanics and mechanotransduction. Ann. Biomed. Eng. 33, 1719–1723. 10.1007/s10439-005-8775-z 16389519

[B77] Theodosis D. T. Poulain D. A. Oliet S. H. (2008). Activity-dependent structural and functional plasticity of astrocyte-neuron interactions. Physiol. Rev. 88, 983–1008. 10.1152/physrev.00036.2007 18626065

[B78] Tweedle C. D. Hatton G. I. (1977). Ultrastructural changes in rat hypothalamic neurosecretory cells and their associated glia during minimal dehydration and rehydration. Cell Tissue Res 181, 59–72. 10.1007/bf00222774 880623

[B79] Tweedle C. D. Smithson K. G. Hatton G. I. (1993). Rapid synaptic changes and bundling in the supraoptic dendritic zone of the perfused rat brain. Exp. Neurol. 124, 200–207. 10.1006/exnr.1993.1190 8287923

[B80] Verbalis J. G. (2010). Brain volume regulation in response to changes in osmolality. Neuroscience 168, 862–870. 10.1016/j.neuroscience.2010.03.042 20417691

[B81] Voisin D. L. Bourque C. W. (2002). Integration of sodium and osmosensory signals in vasopressin neurons. Trends Neurosci. 25, 199–205. 10.1016/s0166-2236(02)02142-2 11998688

[B84] Wang Y.-F. Liu L.-X. Yang H.-P. (2011). Neurophysiological involvement in hypervolemic hyponatremia-evoked by hypersecretion of vasopressin. Transl. Biomed. 2:3. 10:3823/425

[B82] Wang Y.-F. Parpura V. (2016). Central role of maladapted astrocytic plasticity in ischemic brain edema formation. Front. Cell. Neurosci. 10:129. 10.3389/fncel.2016.00129 27242440 PMC4865516

[B85] Wang Y. F. Sun M. Y. Hou Q. Hamilton K. A. (2013a). GABAergic inhibition through synergistic astrocytic neuronal interaction transiently decreases vasopressin neuronal activity during hypoosmotic challenge. Eur. J. Neurosci. 37, 1260–1269. 10.1111/ejn.12137 23406012 PMC3627741

[B86] Wang Y. F. Sun M. Y. Hou Q. Parpura V. (2013b). Hyposmolality differentially and spatiotemporally modulates levels of glutamine synthetase and serine racemase in rat supraoptic nucleus. Glia 61, 529–538. 10.1002/glia.22453 23361961

[B83] Wang Y. F. Zhu H. (2014). Mechanisms underlying astrocyte regulation of hypothalamic neuroendocrine neuron activity. Sheng Li Ke Xue Jin Zhan 45, 177–184. 25219268

[B87] Wilson C. Dryer S. E. (2014). A mutation in TRPC6 channels abolishes their activation by hypoosmotic stretch but does not affect activation by diacylglycerol or G protein signaling cascades. Am. J. Physiol. Renal Physiol. 306, F1018–F1025. 10.1152/ajprenal.00662.2013 24598806

[B88] Wu G. Lu Z. H. Obukhov A. G. Nowycky M. C. Ledeen R. W. (2007). Induction of calcium influx through TRPC5 channels by cross-linking of GM1 ganglioside associated with α5β1 integrin initiates neurite outgrowth. J. Neurosci. 27, 7447–7458. 10.1523/JNEUROSCI.4266-06.2007 17626205 PMC6672619

[B89] Yagil C. Sladek C. D. (1990). Osmotic regulation of vasopressin and oxytocin release is rate sensitive in hypothalamoneurohypophysial explants. Am. J. Physiol. 258, R492–R500. 2309938 10.1152/ajpregu.1990.258.2.R492

[B91] Zeng Y. Tarbell J. M. (2014). The adaptive remodeling of endothelial glycocalyx in response to fluid shear stress. PLoS One 9:e86249. 10.1371/journal.pone.0086249 24465988 PMC3896483

[B90] Zeng J. W. Wang X. G. Ma M. M. Lv X. F. Liu J. Zhou J. G.. (2014). Integrin β3 mediates cerebrovascular remodelling through Src/ClC-3 volume-regulated Cl^−^ channel signalling pathway. Br. J. Pharmacol. 171, 3158–3170. 10.1111/bph.12654 24611720 PMC4080971

[B92] Zhang B. Glasgow E. Murase T. Verbalis J. G. Gainer H. (2001). Chronic hypoosmolality induces a selective decrease in magnocellular neurone soma and nuclear size in the rat hypothalamic supraoptic nucleus. J. Neuroendocrinol. 13, 29–36. 10.1111/j.1365-2826.2001.00593.x 11123513

[B93] Zhao X. Yan X. Liu Y. Zhang P. Ni X. (2016). Co-expression of mouse TMEM63A, TMEM63B and TMEM63C confers hyperosmolarity activated ion currents in HEK293 cells. Cell Biochem. Funct. 34, 238–241. 10.1002/cbf.3185 27045885

[B94] Zhu J. X. Zhu X. Y. Owyang C. Li Y. (2001). Intestinal serotonin acts as a paracrine substance to mediate vagal signal transmission evoked by luminal factors in the rat. J. Physiol. 530, 431–442. 10.1111/j.1469-7793.2001.0431k.x 11158274 PMC2278417

